# Excellent Oxygen Evolution Reaction of Activated Carbon-Anchored NiO Nanotablets Prepared by Green Routes

**DOI:** 10.3390/nano10071382

**Published:** 2020-07-15

**Authors:** Sankar Sekar, Deuk Young Kim, Sejoon Lee

**Affiliations:** 1 Division of Physics & Semiconductor Science, Dongguk University-Seoul, Seoul 04620, Korea; sanssekar@gmail.com (S.S.); dykim@dongguk.edu (D.Y.K.); 2 Quantum-Functional Semiconductor Research Center, Dongguk University-Seoul, Seoul 04620, Korea

**Keywords:** nickel oxide, activated carbon, nanocomposite, electrocatalysts, oxygen evolution reaction

## Abstract

A sustainable and efficient electrocatalyst for the oxygen evolution reaction (OER) is vital to realize green and clean hydrogen production technology. Herein, we synthesized the nanocomposites of activated carbon-anchored nickel oxide (AC-NiO) via fully green routes, and characterized their excellent OER performances. The AC-NiO nanocomposites were prepared by the facile sonication method using sonochemically prepared NiO nanoparticles and biomass-derived AC nanosponges. The nanocomposites exhibited an aggregated structure of the AC-NiO nanotablets with an average size of 40 nm. When using the nanotablets as an OER catalyst in 1 M KOH, the sample displayed superb electrocatalytic performances, i.e., a substantially low value of overpotential (320 mV at 10 mA/cm^2^), a significantly small Tafel slope (49 mV/dec), and a good OER stability (4% decrease of overpotential after 10 h). These outstanding OER characteristics are considered as attributing to the synergetic effects from both the ample surface area of the electrochemically active NiO nanoparticles and the high electrical conductivity of the AC nanosponges. The results pronounce that the fully ecofriendly synthesized AC-NiO nanotablets can play a splendid role as high-performance electrocatalysts for future green energy technology.

## 1. Introduction

Due to both the anxiety of environmental complications and the scarcity of fossil fuels, renewable energy sources have been of vast interest to realize green energy technology [1,2,3]. Hydrogen is one of the most efficient, renewable, and clean energy sources for future eco-energy technology. Among various hydrogen production techniques, the electrocatalytic water electrolysis is a simple and efficient route to produce clean and renewable hydrogen energy [4,5]. In this otherwise powerful technique, there is a crucial fact that the water-splitting efficiency strongly relies on the oxygen evolution reaction (OER) [6]. Recently, Ir/Ru-based oxides are deemed to be a benchmark of the high-performance OER electrocatalysts because of their most active sites and high water-to-hydrogen conversion efficiency [7,8]. Despite such benefits, the tangible applications of Ir/Ru-based oxides have been restricted due to their rapid deactivation, scarcity, and high cost [9,10,11]. Therefore, many researchers have devoted to develop a cheap, stable, and highly efficient OER electrocatalysts. In very recent years, nickel-based conjugates [12,13,14,15,16,17,18,19] and nickel oxide (NiO) nanostructures [20,21,22,23,24,25,26,27,28,29,30,31] have been demonstrated as a promising OER electrocatalyst because of their fast response, high corrosion resistance, earth abundance, low cost, high surface kinetic reaction, and good stability. However, the electrocatalytic OER performances of the NiO nanostructures are still unsatisfiable because of their sluggish kinetics, low electronic conductivity, and limited active sites [32,33]. To release such drawbacks, thus, anchoring of NiO with carbonaceous materials (e.g., carbon particles [32], carbon nanotubes [33,34], carbon fibers [35], carbon nanowires [36], activated carbon (AC) [37], graphene [38,39,40], reduced graphene oxide [41,42], etc.) has been proposed as an effective way to improve the electrical conductivity as well as the vigorous kinetic reaction. For instance, Hoang et al. [37] synthesized Ni/NiO/N-doped AC by using cauliflower leaves, and they showed a low overpotential of 346 mV at 10 mA/cm^2^ and a small Tafel slope of 70 mV/dec. Faisal et al. [40] also reported a low overpotential of 410 mV at 10 mA/cm^2^ when using the Ni/NiO/N-graphene composites as an OER electrocatalyst. Very recently, Mugheri et al. [32] achieved a very low overpotential of 220 mV and a small Tafel slope of 55 mV/dec from NiO/C electrocatalysts. Amongst the various carbonaceous materials, AC is one of the most substantial candidates for synthesizing high-performance AC-NiO nanocomposites because of its large surface area, high conductivity, high durability [43,44,45,46,47]. Furthermore, due to its environmental friendliness, vast abundance, fast regeneration, and low cost, biomass-derived AC has attracted extensive attention from the next-generation energy technology community [43,44,45,46,47]. However, most of all previous works have still used some chemical solutions (e.g., solvents and/or acids) for the formation of both electrocatalysts and carbonaceous materials [20,21,22,23,24,25,26,27,28,29,30,31,32,33,34,35,36,37,38,39,40,41,42,43,44,45,46,47]. Therefore, developing an ecofriendly and chemical-free synthesis method is essential to realize the fully green energy technology.

Motivated by all the above, we investigated the green-synthesis of AC-NiO nanocomposites for obtaining the high-performance OER electrocatalysts. In this article, we report on experimental data for the chemical-free synthesis of AC-NiO nanotablets and their excellent OER characteristics. The nanocomposites were fabricated by the fully ecofriendly-route of the water-assisted sonication by using biomass-derived AC nanosponges and sonochemically-prepared NiO nanoparticles in pure water. The AC-NiO nanotablets showed the remarkable OER performances with a very low overpotential (320 mV at 10 mA/cm^2^ in 1 M KOH) and a considerably small Tafel slope (49 mV/dec). Herein, the formation kinetics, material properties, and electrocatalytic characteristics of the AC-NiO nanotablets are systematically assessed and discussed in detail.

## 2. Experimental Section

### 2.1. Preparation of NiO Nanoparticles

**Figure 1:**
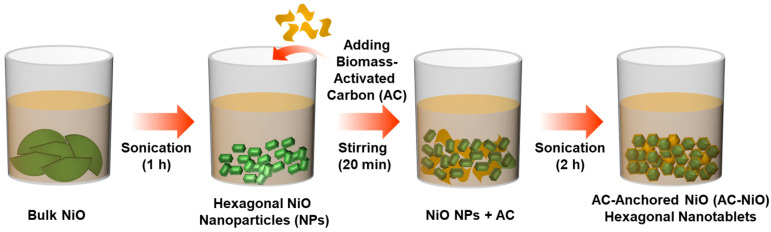
Schematic of the sonochemical procedures for fabricating the NiO nanoparticles and AC-NiO nanotablets.

Figure 1 schematically shows the experimental procedure for the formation of the AC-NiO nanotablets via fully green routes. As an initial task, the NiO nanoparticles were synthesized using commercial bulk NiO (Sigma Aldrich, Seoul, Republic of Korea). First, 1.5 g of bulk NiO powders were liquefied in 80 mL of deionized (DI) water through constant stirring for 15 min. Next, the solution was sonicated for 1 h in a 100 mL glass vessel under high ultrasonic power of 240 W at 35 kHz. Afterward the sonication process, the solution was cleaned, sieved, and dried at 150 °C for 5 h in an electric oven. Then, the greenish NiO nanoparticles were collected.

### 2.2. Derivation of Biomass-AC Nanosponges 

Biomass-AC nanosponges were derived from human hair, which had been collected from Tamil Nadu, India. First, a bundle of human hair was washed in DI water several times, and dried by sunlight for 6 h. After that, the cleaned human hair was burned in an open environment to collect its ashes. Finally, the ashes were carbonized at 300 °C for 2 h in an alumina crucible under nitrogen atmosphere. Through these steps, we were able to obtain the powder type of high-quality biomass-AC nanosponges (Figure S1).

### 2.3. Synthesis of AC-NiO Nanotablets

First, NiO nanoparticles (1.5 g) were dissolved in DI water (80 mL) through the continual stirring step for 15 min. Subsequently, we mixed biomass-AC nanosponges (0.5 g) into the NiO-dissolved solution by stirring for 20 min. Thereafter, the mixture solution was sonicated under high ultrasonic power of 240 W for 2 h at 35 kHz. Finally, the sonicated solution was cleaned, sieved, and dried at 150 °C for 5 h. These processes allowed us to successfully synthesize the AC-NiO nanotablets.

### 2.4. Material Characterizations

The morphological and the compositional properties of the synthesized materials were examined through field-emission scanning electron microscopy (FE-SEM, Inspect-F50, FEI, Mahwah, NJ, USA) and energy dispersive x-ray (EDX) spectroscopy, respectively. In addition, the microstructures of the NiO nanoparticles and the AC-NiO nanotablets were further monitored by transmission electron microscopy (TEM, JEM 2100F, JEOL, Tokyo, Japan). The chemical bonding and the structural characteristics of NiO and AC-NiO were investigated through Raman scattering spectroscopy (LabRAM HR-800, Jobin Yvon, Longjumeau, France) and x-ray diffractometry (XRD, D8-Advance, Bruker, Madison, WI, USA), respectively.

### 2.5. Electrocatalytic Measurements

To fabricate the working electrodes for the OER test, firstly, each active source (i.e., either of NiO or AC-NiO) was mixed with *N*-methyl-2-pyrrolidinone in a separate beaker. Then, each of mixture slurries was coated on the stainless steel substrates (1 cm^2^), and dried at 150 °C for 5 h. We, here, note that the platinum mesh and the saturated calomel electrode (SCE) were used as a counter electrode and a reference electrode, respectively. After fabricating the NiO and the AC-NiO working electrodes, the electrochemical OER performances were examined in a 1 M KOH electrolyte by linear sweep voltammetry (LSV) and cyclic voltammetry (CV) measurements using a three-electrode system (VersaSTAT3, Ametek Scientific Instruments, Berwyn, PA, USA). We, here, note that KOH electrolyte was prepared by using standard grade KOH pellets (Sigma Aldrich, Seoul, Republic of Korea) and DI water. LSV measurements were carried out at the scan rate (r*_s_*) of 1 mV/s in the potential range of 0.1–0.8 V (vs. SCE), and the CV characteristics were evaluated at various scan rates (r*_s_* = 10–100 mV/s) in a potential window of 0–0.5 V (vs. SCE). The chronopotentiometric measurement of the NiO and AC-NiO electrodes were performed at various injection current densities (J_i_) of 10, 20, 30, 40, 50, and 100 mA/cm^2^, respectively. The electrochemical impedance spectroscopy (EIS) measurements were performed at a frequency range of 1 Hz to 10 kHz.

## 3. Results and Discussion

### 3.1. Morphological and Structural Properties of NiO and AC-NiO

**Figure 2:**
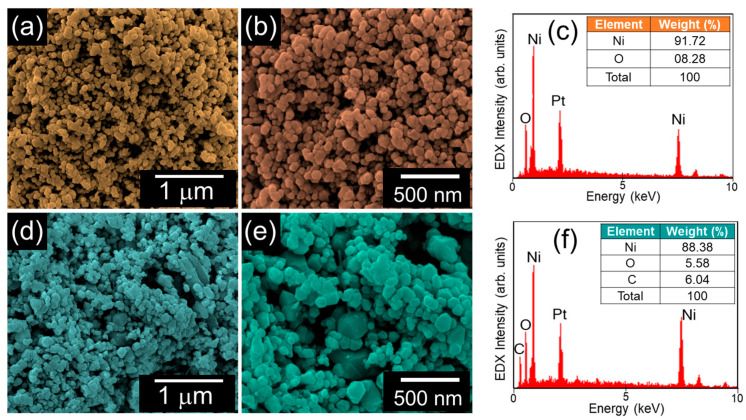
(**a**) Low- and (**b**) high-magnification FE-SEM images of NiO nanoparticles; (**c**) EDX spectrum of the NiO nanoparticles (**d**) low- and (**e**) high-magnification FE-SEM images of the AC-NiO nanotablets; and (**f**) EDX spectrum of the AC-NiO nanotablets.

Figure 2 shows the microstructural and the compositional properties of the NiO nanoparticles and the AC-NiO nanocomposites. From Figure 2a,b one can confirm that the hexagonal NiO nanoparticles are densely packed and interconnected with each other. The NiO nanoparticles contain only their own intrinsic species of Ni and O, except for Pt from conductive-coating for FE-SEM measurements (Figure 2c). For the AC-NiO nanocomposites, the aggregated structures of the nanotablets were visible (Figure 2d,e). In addition, the EDX spectrum confirmed that the AC-NiO nanocomposites obviously involved the C species (Figure 2f). This signifies that the NiO nanoparticles were well-anchored with the AC nanosponges. In order to help understanding the formation kinetics of the AC-NiO nanocomposites via the solvent-free green routes, we here interpret the reaction mechanism of the water-assisted sonication method. For the sonochemical reaction in aqueous solution, water (H_2_O) generates two primary radicals of hydrogen (H^*^) and hydroxyl (OH^*^). Those radicals act as the reductants during sonication of bulk materials [48,49,50,51,52]; hence, bulk NiO (n*NiO*) could be reduced into small NiO nanoparticles (*NiO*_(n)_) under the high ultrasonic power in H_2_O. Such a sonochemical reduction can be expressed as follows:

(1) $$H_{2}O\underset{Sonication}{\to }H^{*}+OH^{*}$$

(2) $$nNiO+H^{*}+OH^{*}\underset{Sonication}{\to }NiO_{\left(n\right)}$$

(3) $$nNiO+AC+H^{*}+OH^{*}\underset{Sonication}{\to }NiO_{\left(n\right)}-AC-NiO_{\left(n\right)}$$

**Figure 3:**
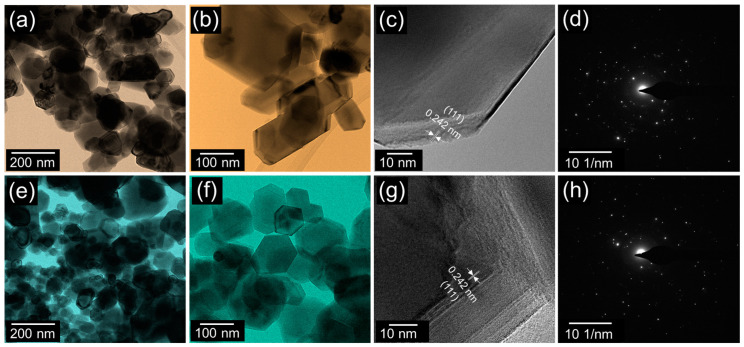
(**a**) Low-magnification TEM image, (**b**) high-magnification TEM image, (**c**) high-resolution TEM image, and (**d**) SAED pattern of the NiO nanoparticles. (**e**) Low-magnification TEM image, (**f**) high-magnification TEM image, (**g**) high-resolution TEM image, and (**h**) SAED pattern of the AC-NiO nanotablets.

To further characterize the prepared materials, we carried out TEM and selective-area electron diffraction (SAED) measurements. As displayed in Figure 3a,b the hexagonal NiO nanoparticles were interconnected with each other, and the average particle size was 75 nm. The interlayer fringe of the nanoparticle was 0.242 nm (Figure 3c), and this value corresponds to the lattice parameter of (111) cubic NiO [53,54]. In addition, the SAED pattern elucidated the NiO nanoparticles to be formed with well-crystallized NiO solid state phases (Figure 3d). Similar to NiO nanoparticles, the AC-NiO nanocomposites were also aggregated by a lot of hexagonal nanotablets (Figure 3e,f). However, the nanocomposites had a smaller average size (40 nm) than that of the pristine NiO nanoparticles because of the additional sonication process for anchoring of AC-NiO. As depicted in the HR-TEM image, the lattice spacing was 0.242 nm (Figure 3g); and the nanotablets still maintained their crystalline phases even after all of the sonochemical processes (Figure 3h).

**Figure 4:**
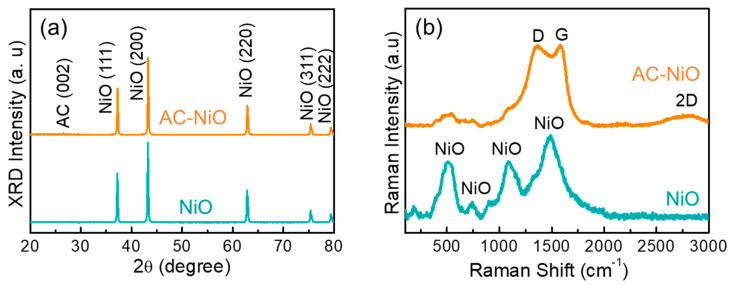
(**a**) XRD patterns and (**b**) Raman spectra of the NiO nanoparticles and the AC-NiO nanotablets.

Figure 4a shows the crystallographic properties of the NiO nanoparticles and the AC-NiO nanotablets. In XRD patterns, both the NiO nanoparticles and the AC-NiO nanotablets exhibited the diffraction peaks of 37.2, 43.2, 62.9, 75.4, and 79.3° from (111), (200), (220), (311), and (222) crystal planes of cubic NiO (JCPDS card no: 04-0835), respectively [55,56]. The only one thing different from each other was that the AC-NiO composites include an additional peak at 26.5°, arising from the (002) phase of AC [45,46,47] (see also Figure S2). This validates that the AC-NiO composites are composed of AC nanosponge-anchored NiO nanoparticles.

Such an aggregated structure can be further clarified from the Raman scattering characteristics of the samples (Figure 4b). In the NiO nanoparticles, four Raman scattering peaks were observable at 510, 742, 1085, and 1490 cm^−1^, and those could be ascribed to the first-order longitudinal optical (LO_1_) phonon mode of the Ni-O lattice vibration, the second-order transverse (TO_2_), the second-order longitudinal optical (LO_2_) phonon modes [39,57], and the two-magnon (2 M) mode of the NiO [31], respectively. Different from the NiO nanoparticles, the AC-NiO nanotablets revealed three additional Raman peaks at 1360, 1586, and 2856 cm^−1^, attributing to D, G, and 2D bands of AC nanosponges [58,59] (see also Figure S3). From the Raman intensity ratio between D and G peaks (I_D_/I_G_ = 0.99), one may also confirm that our biomass-AC was well graphitized [44].

### 3.2. Electrocatalytic Performances of NiO and AC-NiO

**Figure 5:**
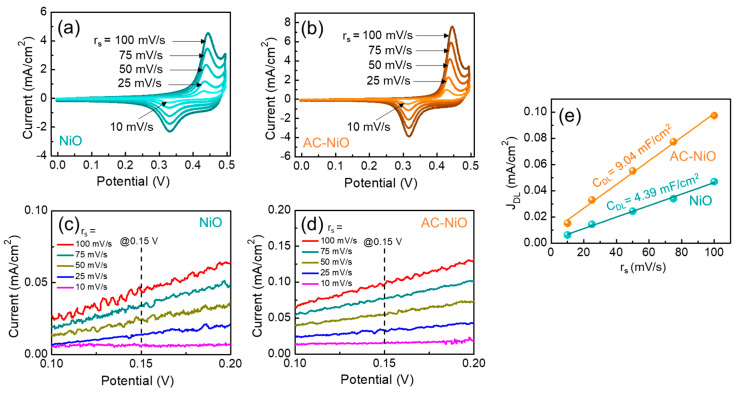
Cyclic voltammetry (CV) curves at various r*_s_* of the oxygen evolution reaction (OER) electrodes composed of the (**a**) NiO nanoparticles and the (**b**) AC-NiO nanotablets. Non-Faradaic current density at various r*_s_* as a function of the potential voltage for the (**c**) NiO nanoparticles and the (**d**) AC-NiO nanotablets. The current density was measured at the non-Faradaic region of 0.1–0.2 V under various r*_s_* (10–100 mV/s). (**e**) J_DL_ as a function of r*_s_*, where the non-Faradaic current density was extracted at the potential voltage of 0.15 V.

The aggregated structure of the AC-NiO nanocomposites could improve the OER performances because of the increases in both the electrochemically active area and the electrical conductivity due to the incorporation of AC. To verify the effect of AC-NiO aggregation, we therefore examined the electrocatalytic characteristics of the samples. Figure 5a,b shows the CV curves at various r*_s_* (10–100 mV/s) for the OER electrodes composed of the NiO nanoparticles and the AC-NiO nanotablets, respectively. Both samples clearly exposed the distinct reduction and oxidation peaks, resulting from the insertion/desertion of electrolyte ions during the anodic/cathodic reaction. Compared to pristine NiO, however, the composite of AC-NiO displays lager integrated CV areas and enhanced current–voltage responses. This validates the AC-NiO nanotablets to possess higher electrical conductivity and large porosity than those of the NiO nanoparticles.

To discriminate such an enhanced OER performance in the AC-NiO composite system, we evaluated the catalytically active site by estimating the electrochemically active surface area (*ECSA*) using the CV data recorded in the linear charging region. From the non-faradaic CV responses in 0.1–0.2 V (Figure 5c,d), the value of *ECSA* can be simply calculated by the following equations [11]:

(4) $$J_{DL}=C_{DL}\times r_{s}/A$$

(5) $$ECSA=C_{DL}/C_{e}$$

where J*_DL_*, C*_DL_*, A, and C*_e_* are the double-layer charging current, the non-Faradic capacitance, the electrode area, and the unit area capacitance of electrolyte (0.04 mF/cm^2^ for KOH), respectively. Figure 5e displays the magnitude of J*_DL_* as a function of r*_s_* at the potential voltage of 0.15 V. The *ECSA* values of NiO and AC-NiO were determined to be 110 and 226 cm^2^, respectively. Compared to NiO, AC-NiO had a lager *ECSA*. Namely, the ion storage and the OER catalytic activity were greater for the AC-NiO nanotablets, compared to the pristine NiO nanoparticles.

**Figure 6:**
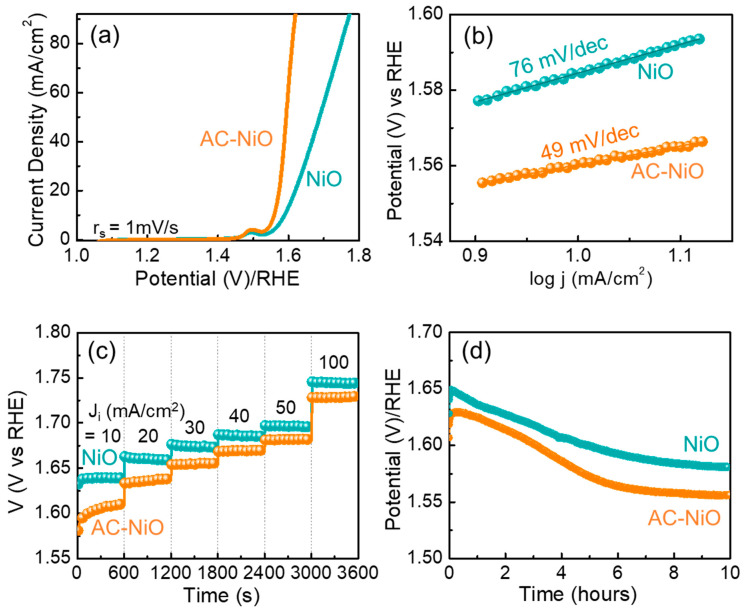
(**a**) Linear sweep voltammetry (LSV) curves (iR corrected), (**b**) Tafel plots, (**c**) multi-current chronopotentiometry under J_i_ = 10–100 mA/cm^2^, and (**d**) OER stability for the NiO nanoparticles and the AC-NiO nanotablets.

Next, we measured the LSV characteristics at r*_s_* = 1 mV/s for the NiO and the AC-NiO OER electrodes. From the iR-corrected LSV curves (Figure 6a), the overpotential (η) of the NiO sample was extracted to be 360 mV at 10 mA/cm^2^, while the AC-NiO sample showed a lower η value of 320 mV at 10 mA/cm^2^.

In addition, the samples revealed the enhanced electrochemical reaction kinetics. Namely, the Tafel slope was determined to be 76 and 49 mV/dec for the NiO and the AC-NiO OER electrodes (Figure 6b), respectively, from the Tafel equation [11]:

(6) $$\eta =s_{T}\log\;\left(J\right)+a$$

Here, *a* and *s_T_* are the fitting parameter and the Tafel slope, respectively. The values of η and *s_T_* were comparable or even much lower than those of earlier OER studies based on Ni and/or NiO (see Table 1). Particularly, the AC-NiO OER electrode shows a very lower overpotential, a higher current, and a smaller Tafel slope. Since those parameters are directly associated with the catalytically active sites and the intrinsic reaction kinetics [20,37,42], we could conjecture that the OER activity was dramatically increased in the AC-NiO OER electrode. In other words, the above results rarified the aforementioned hypothesis that the aggregation of AC-NiO nanocomposites could enhance the OER characteristics due to the increased electrical conductivity and the enlarged electrochemically active area.

Such a superior OER catalytic activity can also affect the chronopotentiometric characteristics. As shown in the chronopotentiometric curves (Figure 6c), the AC-NiO OER electrode exhibited a lower overpotential response at each J_i_ than that of the pristine NiO OER electrode. Furthermore, the AC-NiO OER electrode also showed a better performance in the long-term durability test. As displayed in Figure 6d, the stability slope was more stable for the AC-NiO OER electrode than pristine NiO. Initially, the static voltage profile increased due to the activation of the catalytic reaction; however, the potential value gradually decreased and constantly maintained after few hours because of the stabilization of the electrode material. Namely, NiOOH might be formed on the surface of AC-NiO after few hours of the OER events [33,37], as discussed later. Moreover, the LSV curves were almost identical before and after the 10 h OER stability test (see Figure S4). These signify that the AC-NiO OER electrode could offer a stable electrocatalytic OER activity in KOH medium for a quite long time. After OER stability, we carried out FE-SEM and Raman scattering measurements to further elucidate the microstructural and chemical bonding properties of the electrodes. From FE-SEM measurements, the NiO electrode showed the aggregated structure of the hexagonal nanoparticles (see Figure S5a). However, the AC-NiO electrode still maintained their original nanotablets structure (see Figure S5b). In Raman spectrum (see Figure S6), both the NiO and AC-NiO electrodes exhibited the additional bands at 330 cm^−1^ and 478 cm^−1^, corresponding to the presence of NiOOH [60,61,62]. This corroborates the aforementioned hypothesis that NiOOH was formed on AC-NiO after the OER stability test.

**Figure 7:**
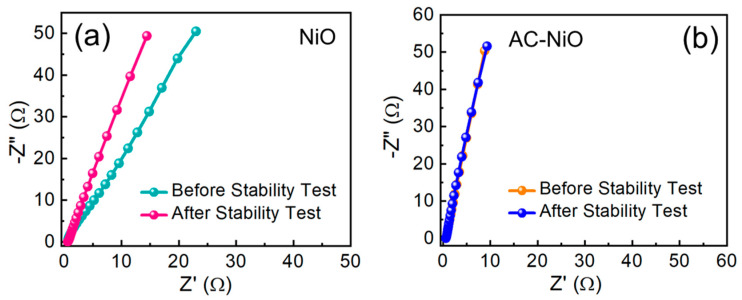
Nyquist plots of the (**a**) NiO nanoparticles and the (**b**) AC-NiO nanotablets before and after the stability test for 10 h.

Finally, the EIS measurements were performed at 1 Hz to 10 kHz to investigate the catalytic kinetic reaction of the electrodes. Figure 7a,b displays the Nyquist plots of the NiO and the AC-NiO OER electrodes, respectively. In both cases, the EIS spectra exhibited a linear feature in the low frequency region because of the dispersion of electrolyte within the electrode. Here, it should be noticeable that no semicircles were observable in the high frequency region. Since the semicircle was related to the charge transfer resistance [38,42] as well as the series resistance, the absence of the semicircle presents the decreases in both the electronic and the ionic resistance in the electrochemical scheme [63,64]. For the NiO and the AC-NiO OER electrodes, the series resistance values were determined to be 2.16 and 1.57 Ω, respectively. Therefore, we could conclude that the enhanced OER performances originated from the higher electrical conductivity and the lower ionic resistance in the AC-NiO nanocomposite system.

## 4. Summary and Conclusions

The nanocomposites of the aggregated AC-NiO nanotablets were effectively synthesized via fully green and facile procedures by using biomass-AC nanosponges and sonochemically prepared NiO nanoparticles through water-assisted sonication. When using the AC-NiO nanotablets as an OER electrode source material, we obtained a very low overpotential of 320 mV at 10 mA/cm^2^ in 1 M KOH electrolyte. In addition, the sample clearly showed a very small Tafel slope of 49 mV/dec, and exhibited an excellent stability. The results imply that the AC-NiO nanocomposites hold great potential as an excellent electrocatalyst material for high-performance green-energy technology.

## Figures and Tables

**Table 1:** Comparison of OER activity of NiO and AC-NiO with previously reported nickel and nickel oxide-based electrocatalysts.

Catalyst	Current Density (mA/cm2)	Overpotential η (mV)	Tafel Slope (mV/dec)	Electrolyte	Reference
AC-NiO	10	320	49	1 M KOH	This work
NiO	10	360	79	1 M KOH	This work
NiO/C	10	220	55	1 M KOH	[32]
NiO-CNT	10	301	82	1 M KOH	[34]
Ni-NiO-CNT	10	320	80	1 M KOH	[33]
Co_3_O_4_@NiO	10	330	101	1 M KOH	[26]
NiO/Ni-350	10	345	53	1 M KOH	[30]
NiO_x_-AC-500	10	346	70	0.1 M KOH	[37]
NiO@Ni/WS_2_/CC	50	347	108.9	1 M KOH	[27]
NiCo	10	367	40	1 M KOH	[18]
Ni/P-C	10	368	67	0.1 M KOH	[17]
NiO-300	10	370	156	1 M KOH	[25]
Ni@NiO/N–C	10	390	100	1 M KOH	[36]
β-Ni(OH)_2_	10	415	60	1 M KOH	[29]
NiCo_2_O_4_/CNTs	10	416	68	1 M KOH	[19]
NiO/Ni	10	440	91	1 M KOH	[28]
Ni/NiO@rGO	10	480	41	0.5 M KOH	[41]

## Supplementary Materials

The following are available online at https://www.mdpi.com/2079-4991/10/7/1382/s1, Figure S1: (a) FE-SEM and (b) TEM image of the AC nanosponges; Figure S2: XRD pattern of the AC nanosponges; Figure S3: Raman spectra of AC nanosponges; Figure S4: LSV curves of (a) NiO and (b) AC-NiO electrodes for OER before and after the stability test; Figure S5: FE-SEM images of (a) NiO and (b) AC-NiO after the stability test; Figure S6: Raman spectra of (a) NiO and (b) AC-NiO after the stability test.
