# Supplementary material for: Excellent Oxygen Evolution Reaction of Activated Carbon-Anchored NiO Nanotablets Prepared by Green Routes
Source: Nanomaterials (Basel). 2020 Jul 15;10(7):1382. doi: 10.3390/nano10071382 (PMC7408599; doi:10.3390/nano10071382)
Supplement: Supplementary file 1 [file nanomaterials-10-01382-s001.pdf]

nanomaterials

Supplementary Materials

Excellent Oxygen Evolution Reaction of Activated Carbon-Anchored NiO Nanotablets Prepared by Green Routes

Sankar Sekar [1,2], Deuk Young Kim [1,2] and Sejoon Lee [1,2,*]

1 Division of Physics & Semiconductor Science, Dongguk University-Seoul, Seoul 04620, Korea; sanssekar@gmail.com (S.S.); dykim@dongguk.edu (D.Y.K.)
2 Quantum-functional Semiconductor Research Center, Dongguk University-Seoul, Seoul 04620, Korea
* Correspondence: sejoon@dongguk.edu; Tel.: +82-2-2260-3946

Morphological Properties of Biomass-AC

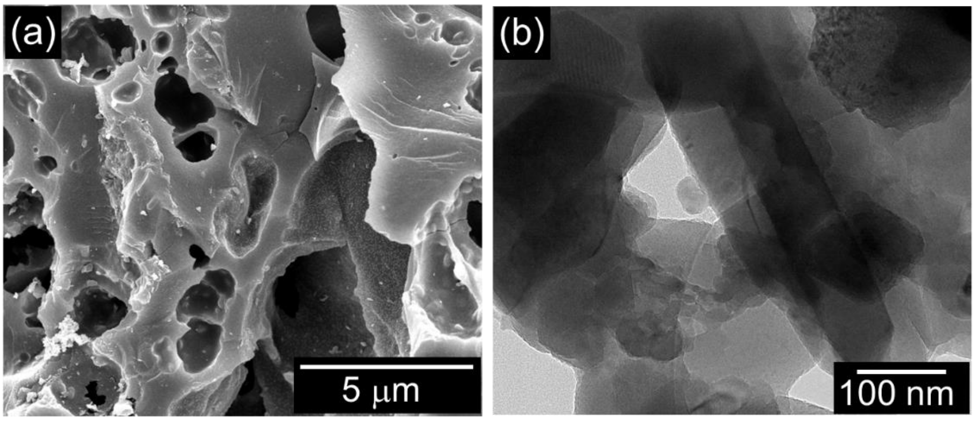

Figure S1. (**a**) FE-SEM and (**b**) TEM image of the AC nanosponges.

Structural Properties of Biomass-AC

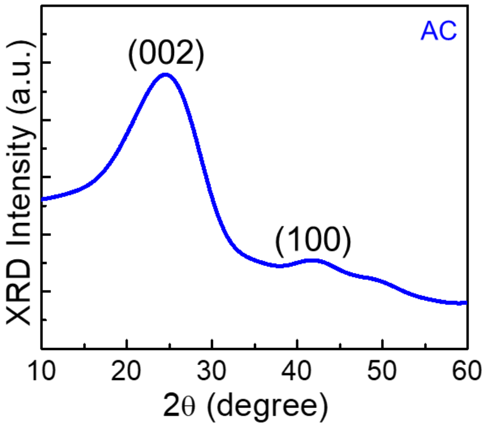

Figure S2. XRD pattern of the AC nanosponges.

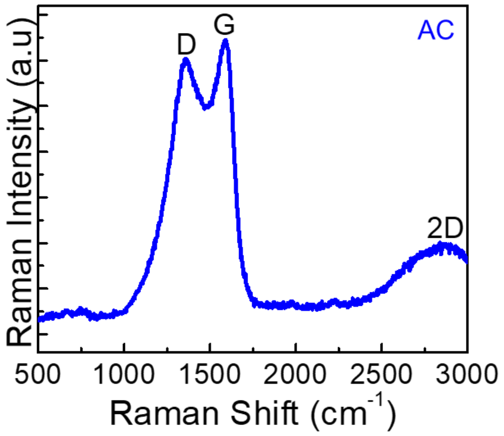

Figure S3. Raman spectra of AC nanosponges.

Electrocatalytic Properties of NiO and AC-NiO

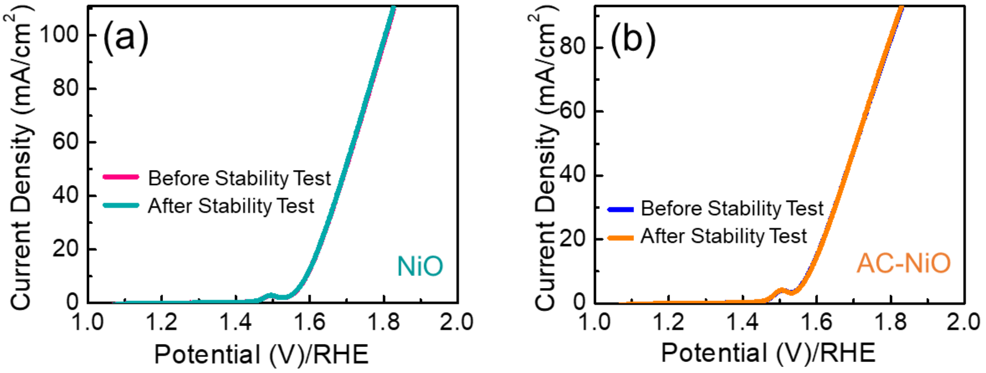

Figure S4. LSV curves of (**a**) NiO and (**b**) AC-NiO electrodes for OER before and after the stability test.

Morphological Properties of NiO and AC-NiO Electrodes After Stability Test

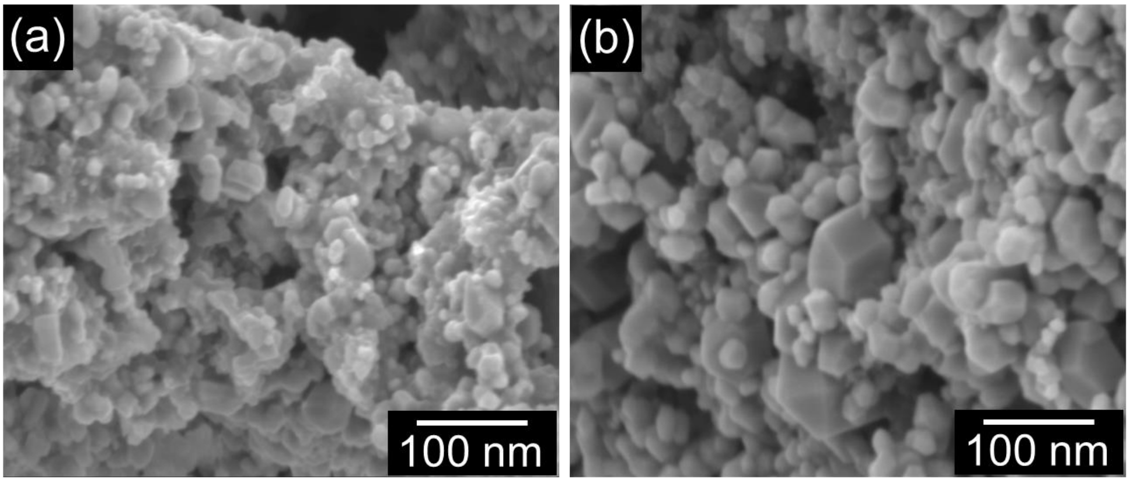

Figure 5S. FE-SEM images of (**a**) NiO and (**b**) AC-NiO after the stability test.

Structural Properties of NiO and AC-NiO Electrodes After Stability Test

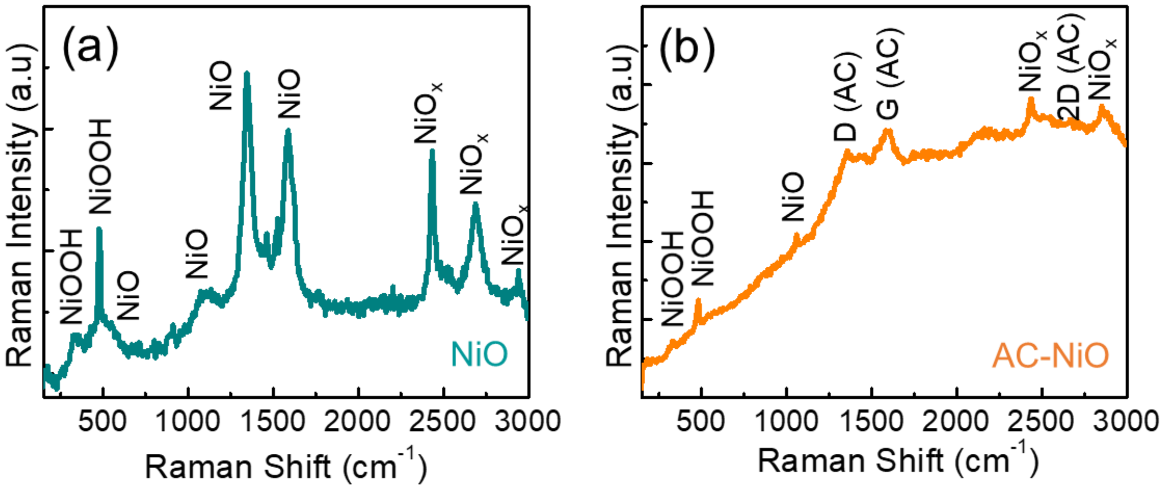

Figure S6. Raman spectra of (**a**) NiO and (**b**) AC-NiO after the stability test.
